# *Wolbachia* surface protein induces innate immune responses in mosquito cells 

**DOI:** 10.1186/1471-2180-12-S1-S11

**Published:** 2012-01-18

**Authors:** Sofia B Pinto, Mara Mariconti, Chiara Bazzocchi, Claudio Bandi, Steven P Sinkins

**Affiliations:** 1 Peter Medawar Building for Pathogen Research &NDM Experimental Medicine / Dept. Zoology, University of Oxford, Oxford, UK; 2 Sezione di Patologia Generale e Parassitologia, DIPAV, Università di Milano, Milan, Italy

## Abstract

**Background:**

*Wolbachia* endosymbiotic bacteria are capable of inducing chronic upregulation of insect immune genes in some situations and this phenotype may influence the transmission of important insect-borne pathogens. However the molecules involved in these interactions have not been characterized.

**Results:**

Here we show that recombinant *Wolbachia* Surface Protein (WSP) stimulates increased transcription of immune genes in mosquito cells derived from the mosquito *Anopheles gambiae*, which is naturally uninfected with *Wolbachia*; at least two of the upregulated genes, *TEP1* and *APL1*, are known to be important in *Plasmodium* killing in this species. When cells from *Aedes albopictus*, which is naturally *Wolbachia*-infected, were challenged with WSP lower levels of upregulation were observed than for the *An. gambiae* cells.

**Conclusions:**

We have found that WSP is a strong immune elicitor in a naturally *Wolbachia*-uninfected mosquito species (*Anopheles gambiae*) while a milder elicitor in a naturally-infected species (*Aedes albopictus*). Since the WSP of a mosquito non-native (nematode) *Wolbachia* strain was used, these data suggest that there is a generalized tolerance to WSP in *Ae. albopictus*.

## Background

*Wolbachia pipientis* is a maternally inherited endosymbiotic bacterium that infects a wide range of nematodes and arthropods. It is responsible for the induction of several forms of reproductive manipulation in its arthropod hosts, all of which favour infected females at the expense of their uninfected counterparts. Cytoplasmic incompatibility, classically seen in its unidirectional form in crosses between uninfected females and infected males where there is high embryo mortality, provides a powerful insect population invasion capacity. Recently, the presence of *Wolbachia* has been associated with the inhibition of viral [1-5] filarial nematode [6] and *Plasmodium *[3,7] pathogens. In addition, *Wolbachia* is capable of inducing the production of anti-oxidant enzymes and reactive oxygen species (ROS) [8], innate immune effectors [6,7,9] as well as increasing haemocyte densities [10]. However the molecular nature of the interactions between this symbiotic bacterium and the insect immune system are not well characterized. If *Wolbachia* is to be used optimally in applied strategies to disrupt pathogen transmission in mosquitoes and other pest insects, it is important to gain a better understanding of what *Wolbachia* molecules are involved in eliciting insect immune responses, and whether responses to these molecules differ between naturally *Wolbachia*-infected and uninfected hosts.

*Wolbachia* and its products have been shown to evoke strong innate immune responses in mammals and are very important in establishing and augmenting inflammatory pathogenesis of the diseases caused by filarial nematodes [11-13]. In particular the *Wolbachia* Surface Protein (WSP) has been shown to elicit innate immune induction via TLR2 and TLR4 activation in both humans and mice [14] and to inhibit apoptosis in neutrophils through inhibition of caspase-3 activity [15].

In this study we investigated whether WSP can also induce innate immune responses in insects, using mosquito cell lines originating from both naturally *Wolbachia*-uninfected and *Wolbachia*-infected mosquito species. An additional aim was to identify PAMPs (pathogen associated molecular patterns) that can elicit strong immune responses in mosquitoes, which could be useful for novel disease control strategies; thus in order to avoid the complications of possible strain-host co-adaptations, we have initially used WSP derived from a nematode *Wolbachia* rather than from an insect-derived *Wolbachia* strain. 

## Results

###  WSP is a strong innate immune response elicitor in *An. gambiae* cells*.*

**Figure 1:**
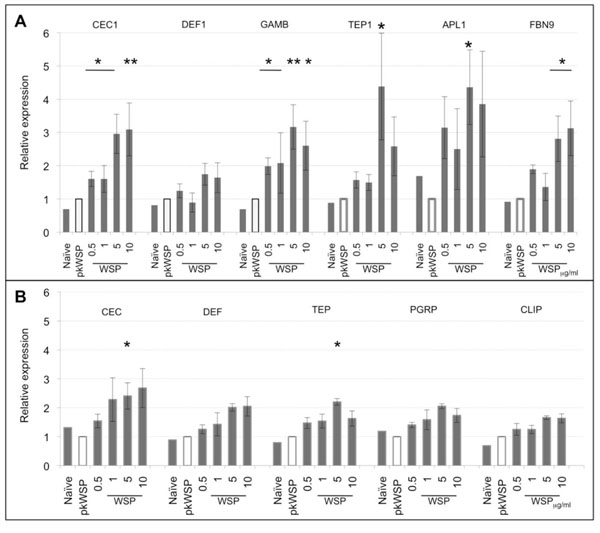
**WSP challenge in mosquito cells.** qRT-PCR analysis of AMPs and innate immune genes at 3h post-WSP challenge in 4a3A (A) and Aa23T (B). Increased expression dependent on WSP quantities up to 5μg/ml was detected in all genes tested. Relative expressions were calculated to pkWSP (WSP protein treated with proteinase K) challenged cells and represent the average of 4 biological repeats +/- SE. Statistical analysis where performed using a Wilcoxon rank sum test (*p<0.05, **p<0.01).

In the *An. gambiae* cells, the antimicrobial peptide-encoding genes *Cecropin 1* (*CEC1*) and *Gambicin* (*GAMB*) showed elevated levels of transcription in the presence of WSP compared to negative controls (naïve and proteinase K-treated-pkWSP) [14] and responded in a dosage dependent fashion, when different concentrations of WSP up to 5μg/ml were used (Fig1A). Their mRNA levels were increased in the presence of WSP to similar degrees and statistically significant differences were observed for all WSP quantities used. In contrast, *Defensin 1* (*DEF1*) which has been shown to be primarily active against Gram-positive bacteria [16], showed only a small degree of upregulation that was not statistically significant. Increased concentrations of WSP also increased the transcription levels of complement-like gene *TEP1*, *Anopheles Plasmodium-responsive Leucine-rich repeat 1* (*APL1*) and *Fibrinogen 9* (*FBN9*) (Fig1A). In comparison to the AMPs, *TEP1* and *APL1* showed a higher induction level with respectively 4 and 5-fold peaks. Significant upregulation was also seen at a concentration of 5μg/ml of WSP for all three genes (p<0.05). This data suggests that in this naturally *Wolbachia*-uninfected mosquito species, WSP is capable of inducing the transcription of innate immune factors such as AMPs, complement-like proteins and fibrinogen genes, all of which are involved in anti-parasitic responses in *An. gambiae*. 

### WSP is a mild innate immune response elicitor in *Ae. albopictus* cells

We next examined whether WSP has the same capacity to elicit an immune response in a species that naturally harbours *Wolbachia*. Uninfected *Ae. albopictus* Aa23 cells [17] were challenged with WSP and transcription level of immunity genes monitored as for the *An gambiae* cell line. All genes tested showed elevation in mRNA levels with increased WSP concentration up to 5μg/ml (Fig1B), but these were less pronounced when compared to the 4a3A cell line*.* Statistically significant upregulation was seen only for CEC and TEP when 5μg/ml WSP was used (p<0.05, Fig1B).

### Only early phase induction is seen after WSP challenge in both cell lines

**Figure 2:**
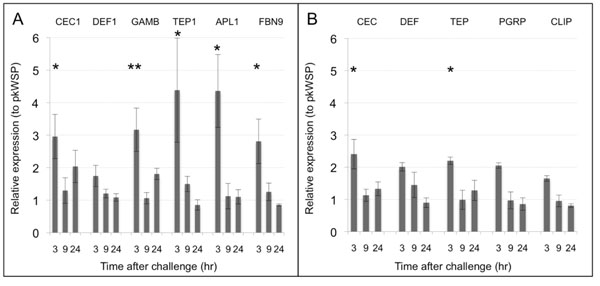
**Dynamics of WSP challenge in mosquito cells.** qRT-PCR analyses in 4a3A **(A)** and Aa23T **(B)** cell lines at 3, 9 and 24h after WSP challenge detect significant upregulation for all tested genes at 3h post-challenge. With the exception of *CEC1* and *GAMB*, mRNA levels return back to control levels at 24h. Relative expressions were calculated to pkWSP-challenged cells and represent the average of 4 biological repeats +/- SE. Statistical analysis where performed using Wilcoxon Rank Sum Test (*p<0.05, **p<0.01).

Innate immune response activation is commonly divided into early phase response (2-4hr post challenge) and late phase response (24hr post challenge), and so far we have shown that WSP can be a strong PAMP at this early phase response (3h post challenge). To determine the dynamics of this immune response, both cell lines were stimulated with 5μg/ml and monitored at 3, 9 and 24h post challenge. In the 4a3A cell line all innate immune transcription is shut down at 9h post infection. For only *CEC1* and *GAMB* a mild induction (2-fold) at 24hr post challenge was detected, however this induction was not statistically significant (Fig2A). In the case of Aa23T cell line immune activation is decreased back to basal levels at 9hr post infection and no late phase induction was detected.

### The *Ae. albopictus* cells are capable of mounting a strong immune response

**Figure 3:**
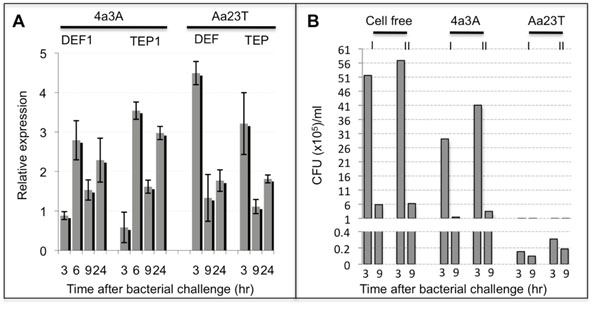
**4a3A and Aa23T immune response to bacterial challenges. (A)** qRT-PCR analysis at 3, 6, 9 and 24h after cell challenge with a mixture of heat-killed *E. coli* and *E. faecalis* show both early and late phase induction of *DEF* and *TEP* in both mosquito species. The time of early phase induction varies between species. Upregulation levels for each gene are similar between the two cell lines. Relative expressions were calculated to PBS-challenged cells and represent the average of 3 biological repeats +/- SE **(B)** 99% of *E. coli* is rapidly cleared by Aa23T cell line at 3h post-infection while for 4a3A only about 14% have been killed when compared to the same amount of bacteria incubated in cell-free (CF) medium. The starting amount used in each case was 25µl per well of culture with an OD600 reading of 0.05, which represents approximately 15-18M CFU/ml. I -Set I; II -Set II.

To exclude the possibility that the differences observed between these cell lines may be due to an impaired immune response in the particular *Ae. albopictus* line used, the responses of both cell lines to bacterial challenge and their capacity to clear a live bacterial infection was tested. Both cell lines were challenged with a mixture of heat-killed *Escherichia coli* and *Enterococcus faecalis*, and relative transcription monitored from 3-24h as above. In the 4a3A cell line peak immune induction of both *DEF1* and *TEP1* was seen at 6h rather than 3h, which for *DEFD* and *TEP* in Aa23T line already showed strong transcription levels. When looking at the peak levels of upregulation, in Aa23T cell line *DEFD* and *TEP* levels reach 4.5 and 3-fold respectively, while *DEF1* and *TEP1* show 3-3.5-fold levels in the 4a3A cell line (Fig3A). To test for the capacity of each cell line to clear an *E. coli* infection, live *E. coli*- TET^r^ was added to 3h conditioned cell culture. Cell medium was collected at 3 and 9h post *E. coli* addition, diluted in LB-TET medium and plated on LB-TET plates. Colony forming units (CFU) where counted for several dilutions for each condition. The Aa23T cells at 3h post-*E coli* addition had cleared 99% of bacteria from the culture medium in comparison with only 14% of bacteria cleared in 4a3A cell culture when compared to the same amount of bacteria incubated in cell-free (CF) medium.

## Discussion

Obtaining a better understanding of *Wolbachia*-host immune interactions in insects is particularly important at the current time given the recently described effects of *Wolbachia* in inhibiting the development or dissemination of several very important mosquito-borne human pathogens. This study shows that, as previously observed using mammalian cells, the *Wolbachia* WSP protein is a potent innate immune elicitor in insects. The responses between the two mosquito cell lines to WSP challenge are mechanistically similar: 1) they are dosage dependent, increasing with increasing amounts of WSP up to 5μg/ml; 2) peak induction is seen at 5μg/ml, while higher concentrations sometimes reduce the mRNA levels; and 3) the immune gene transcription was at a maximum at 3h post challenge (early phase induction) and do not show late phase induction. The major difference is the level of upregulation between the two species: detected peak induction of 3 to 5-fold in the naturally *Wolbachia*-uninfected cell line compared to just 2-fold induction in the naturally infected one. Tolerance effects due to previous natural *Wolbachia* exposure have been described [18] and seem likely to be contributing to the differences observed between these cell lines in their response to WSP. The control experiments also show that Aa23T can show strong induction of immune gene transcription and can effectively clear a bacterial infection. Thus the differences seen between WSP-associated immune induction between these cell lines are not due to impaired immune responses in Aa23T.

In this experimental set-up the WSP protein will be extracellular, and although *Wolbachia* itself is mostly located within intracellular vacuoles in insects, bacterial protein will be released into the hemocele, for example through regular cellular turnover / apoptosis. This mirrors the situation in humans where WSP elicits antibody responses in lymphatic filariasis patients despite *Wolbachia* itself being located inside vacuoles within the filarial nematodes [19]. In the insect hemocele WSP has the potential to elicit innate immune responses from hemocyte immune cells, and the same applies in these cell lines.

Further studies of insect immune responses to WSP may include the examination of levels of immune response to intracellular WSP, using transformation / transfection studies (although these will not exactly replicate the intra-vacuole localization of *Wolbachia* itself). Furthermore, the possibility of different levels of immune response to WSP derived from various insect *Wolbachia* strains can be examined, particularly in the case of the *Ae. albopictus* cells which are derived from a naturally *Wolbachia*-infected species and could thus show varying degrees of tolerance to different WSP molecules. These basic biology questions are also relevant to the important applied aim of identifying potent PAMPs that might be incorporated in transgenic strategies to ‘prime’ the mosquito immune system, and thus impair pathogen transmission. The *Dirofilaria Wolbachia*-derived WSP used here appears to hold potential in this respect, since it induces the upregulation of genes (particularly *TEP1* and *APL1*) that are directly involved in *Plasmodium* killing in *Anopheles* mosquitoes.

## Conclusions

Similarly to mammals, the major surface protein of the endosymbiotic bacteria *Wolbachia* (WSP) can induce strong innate immune responses in insects at the transcriptomic level. Antimicrobial peptides as well as important immune effector genes are up-regulated when recombinant WSP is used to challenge mosquito cell lines. Interestingly the response between a naturally-uninfected mosquito and a naturally -infected mosquito is qualitatively similar but quantitatively distinct. The *Wolbachia* naïve host is capable of mounting a very strong upregulation to WSP as opposed to the *Wolbachia* cleared host suggesting that tolerance effects due to previous *Wolbachia* exposure may be contributing to this particular phenotype. 

## Methods

### Cell cultures

Two cell lines were used: 4a3A derived from the naturally *Wolbachia*-uninfected mosquito species *Anopheles gambiae *[20] and Aa23 from the naturally *Wolbachia*-infected mosquito species *Aedes albopictus *[17]. *w*AlbB-strain infection present in Aa23 was cured via Tetracycline treatment (100μg/ml) for 5 days. *Wolbachia* absence after drug treatment was confirmed using PCR and the derived cell line was subsequently called Aa23T. Cell lines were maintained at 27 °C and grown in Schneider medium (Promo Cell) supplemented with 10% heat-inactivated FCS, 1% penicillin-streptomycin (Gibco). 

### WSP and bacterial cell challenges

Prior to cell challenges, cultures were re-suspended in growth medium and counted using a heamocytometer. For all experiments, approximately 2million cells were seeded per well in 6-well plates. Varying concentrations (0.5-10μg/ml) of stringently purified endotoxin-free recombinant WSP, obtained from the nematode *Dirofilaria immitis *[14,19], were used to challenge the cells. Proteinase k-treated WSP (pkWSP) [14,19] was used at a concentration of 5μg/ml.

Logarithmic phase cultures of *E. coli* and *E. faecalis* were washed three times in PBS and re-suspended in Hank-balanced salt solution (Sigma) at OD (A_600_ nm) of 0.4 prior to heat inactivation at 80 C. For challenge, 30 μl of a 1:1 mixture of heat killed *E. coli* and *E. faecalis* were used per well. Logarithmic phase cultures of *E. coli* K12 TET^r^ strain (NEB) were washed and re-suspended in PBS to a final OD (A_600_ nm) of 0.05. For challenge, 25 μl of the bacterial culture was added to 3hr conditioned cell culture or 3hr incubated Schneider medium (cell-free). Cell medium was collected at 3 and 9hr post *E. coli* addition, plated in serial dilutions onto LB-TET agar plates and the next day the number of CFUs was determined. 

### RNA isolation, cDNA synthesis and real-time quantitative reverse transcription PCR (qRT-PCR)

Total RNA was isolated using TRIzol reagent (Invitrogen) and DNAseI (NEB) treated. First strand cDNA syntheses were performed in a 10μl reaction volume with 1-1.5μg of total RNA using the High Capacity RNA-to-cDNA kit (Applied Biosystems). Real-time quantitative reverse transcription PCR (qRT-PCR) amplifications were performed with Express SYBR GreenER PCR mastermix (Invitrogen) and analyzed using the Chromo4^TM^ detection system (Bio-Rad) following manufacturer’s instructions. Expression levels were calculated by the relative standard curve method, as described in Technical bulletin #2 of the ABI Prism 7700 Manual (Applied Biosystems), using as an endogenous reference ribosomal proteins S7 and L17 for *An. gambiae* and *Ae. albopictus* cell lines, respectively. pkWSP was used as the exogenous calibrator in all experiments. Primers were designed using Geneious^TM^ software (Biomatters Ltd) and sequences are listed in Table1. Data from 4 independent biological repeats was analysed with a Wilcoxon rank of sum test.

**Table 1:** Primers used in qRT-PCR

	Forward primer	Reverse primer
***An gambiae***		
APL1	ACCAGCCGCAGTTTGATAG	CAATCCCAGTCATTATGCGA
RpS7^*^, *CEC1*, *DEF1 ref *[21]and *GAMB*, *TEP1*, *FBN9 ref *[*22*]		
***Ae albopictus***^!^		
DEF (D) ^*^	TTCGATGAACTACCGGAGGA	AGCACAAGCACTGTCACCAA
RpL17^*^	AGTGCGTTCCATTCCGTC	CTTCAGCGTTCTTCAACAGC
*CEC* (*A1*), *TEP* (*20*), *PGRP* (*SP1*) *and CLIP* (*B37*)* ref *[*23*]		

*RpS7 was used as the reference gene in *An. gambiae* analysis while RpL17 was the reference for *Ae. albopictus*. ^!^The *Ae albopictus* immune gene primers have been determined via degeneracy against the corresponding *Ae. aegypti* orthologous genes shown in brackets.

## Authors' contributions

SBP participated in the design of the study, carried out experimental work, data analysis and drafted the manuscript. MM carried out experimental work and data analysis. CB provided reagents and experimental support. ClB participated in the design of the study and helped draft the manuscript. SPS participated in the design of the study, provided reagents and drafted the manuscript. All authors read and approved the final manuscript.

## Competing interests

The authors declare that they have no competing interests.
